# The Combined Effect of Branching and Elongation on the Bioactivity Profile of Phytocannabinoids. Part I: Thermo-TRPs

**DOI:** 10.3390/biomedicines9081070

**Published:** 2021-08-23

**Authors:** Daiana Mattoteia, Aniello Schiano Moriello, Orazio Taglialatela-Scafati, Pietro Amodeo, Luciano De Petrocellis, Giovanni Appendino, Rosa Maria Vitale, Diego Caprioglio

**Affiliations:** 1 Dipartimento di Scienze del Farmaco, Università del Piemonte Orientale, Largo Donegani 2, 28100 Novara, Italy; daiana.mattoteia@uniupo.it (D.M.); giovanni.appendino@uniupo.it (G.A.); 2 Institute of Biomolecular Chemistry, National Research Council (ICB-CNR), Via Campi Flegrei 34, 80078 Pozzuoli, Italy; aniello.schianomoriello@icb.cnr.it (A.S.M.); pamodeo@icb.cnr.it (P.A.); luciano.depetrocellis@icb.cnr.it (L.D.P.); 3 Epitech Group SpA, Saccolongo, 35100 Padova, Italy; 4 Dipartimento di Farmacia, Università di Napoli Federico II, Via Montesano 49, 80131 Napoli, Italy; scatagli@unina.it

**Keywords:** phytocannabinoids, cannabichromene, thermos-TRPs, TRPA1, α,α-dimethylheptyl effect

## Abstract

The affinity of cannabinoids for their CB_1_ and CB_2_ metabotropic receptors is dramatically affected by a combination of α-branching and elongation of their alkyl substituent, a maneuver exemplified by the *n*-pentyl -> α,α-dimethylheptyl (DMH) swap. The effect of this change on other cannabinoid end-points is still unknown, an observation surprising since thermo-TRPs are targeted by phytocannabinoids with often sub-micromolar affinity. To fill this gap, the α,α-dimethylheptyl analogues of the five major phytocannabinoids [CBD (**1a**), Δ^8^-THC (**6a**), CBG (**7a**), CBC (**8a**) and CBN (**9a**)] were prepared by total synthesis, and their activity on thermo-TRPs (TRPV1-4, TRPM8, and TRPA1) was compared with that of one of their natural analogues. Surprisingly, the DMH chain promoted a shift in the selectivity toward TRPA1, a target involved in pain and inflammatory diseases, in all investigated compounds. A comparative study of the putative binding modes at TRPA1 between DMH-CBC (**8b**), the most active compound within the series, and CBC (**8a**) was carried out by molecular docking, allowing the rationalization of their activity in terms of structure–activity relationships. Taken together, these observations qualify DMH-CBC (**8b**) as a non-covalent TRPA1-selective cannabinoid lead that is worthy of additional investigation as an analgesic and anti-inflammatory agent.

## 1. Introduction

**Figure 1:**
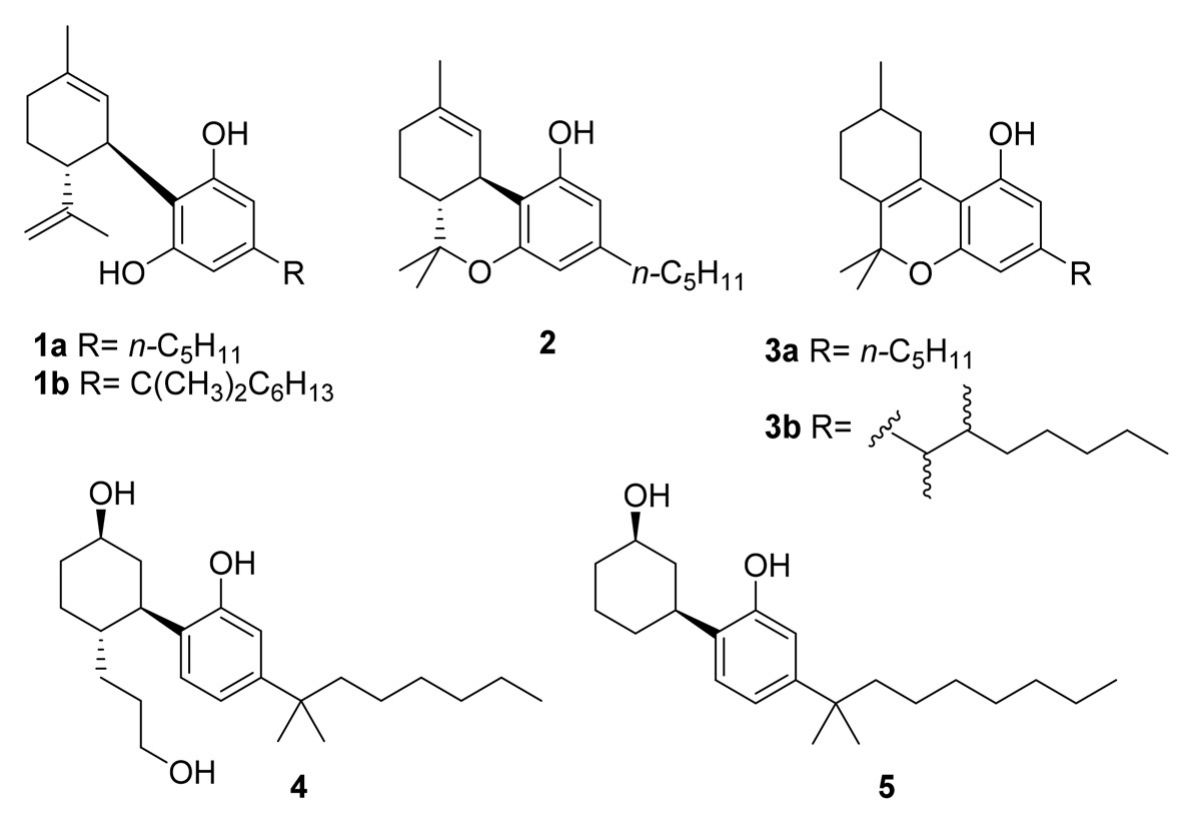
Structures of CBD (**1a**), its α,α-dimethylheptyl analogue (**1b**), Δ^9^-THC (2), Δ**^6a^**^,**10a**^ THC (**3a**), pyrahexyl (**3b**), CP-55.940 (**4**), and cannabicyclohexanol (**5**).

Phytocannabinoids from cannabis (*Cannabis sativa* L.) are characterized by a linear alkyl substituent spanning one to six carbons bound to their resorcinol core, with the pentyl substitution being by far the most common one [1]. The existence of branched chain cannabinoids of the *iso*- and *ante-iso* series is, in principle, plausible from a biogenetic standpoint, since the alkylresorcinol moiety of phytocannabinoids is of ketide derivation, and branching would therefore simply require the replacement of the acetate-derived starter with one derived from a branched amino acid. However, branched phytocannabinoids of this type have only been tentatively detected as trace constituents of cannabis, largely remaining unconfirmed curiosities in its inventory of constituents [1]. Conversely, non-biogenetic branching at the benzyl carbon of the alkyl residue has played a critical role in research on cannabis and cannabinoids ever since the early synthesis of these compounds by Adams in the early forties of the past century [2]. Adams was unsuccessful in the isolation of the narcotic principle of cannabis, but the generation of a mixture of narcotic tetrahydrocannabinols from the acidic treatment of cannabidiol (CBD, Figure 1, **1a**) made him correctly postulate a structure of this type for this elusive principle, whose structure was eventually established as **2** by Mechoulam two decades later [3]. 

In the course of these studies, Adams discovered that Δ**^6a^**^,**10a**^-tetrahydrocannabinol (**3a**), an unnatural compound relatively easily available by total synthesis, could replicate most of the activity of the elusive narcotic principle of cannabis, and investigated the activity of analogues where the *n*-pentyl substituent of the model compound was replaced by longer, shorter, and branched alkyl groups [2]. During these studies, the remarkable potency of compounds bearing substituents on the benzyl and the homobenzyl positions of the alkyl group was discovered. One of these compounds, named pyrahexyl (**3b**) [4], was next developed as an incapacitating non-lethal war weapon and as anti-riot agent, but these studies terminated when the US signed the convention on the ban of chemical weapons in the early sixties [2]. A decade later, capitalizing on these observations, Pfizer chemists developed a series of ultra-potent and less lipophilic analogues of Δ^9^-tetrahydrocannabinol (**2**) that proved instrumental in identifying the two cannabinoid receptors, and that are still used today as a reference for cannabinoid activity, as exemplified by CP-55,940 (**4**). Unfortunately, these compounds are also popular designer drugs for herbal incenses (spices), as shown by cannabicyclohexanol (**5**), the first synthetic cannabinoid discovered in this type of product [4].

**Figure 2:**
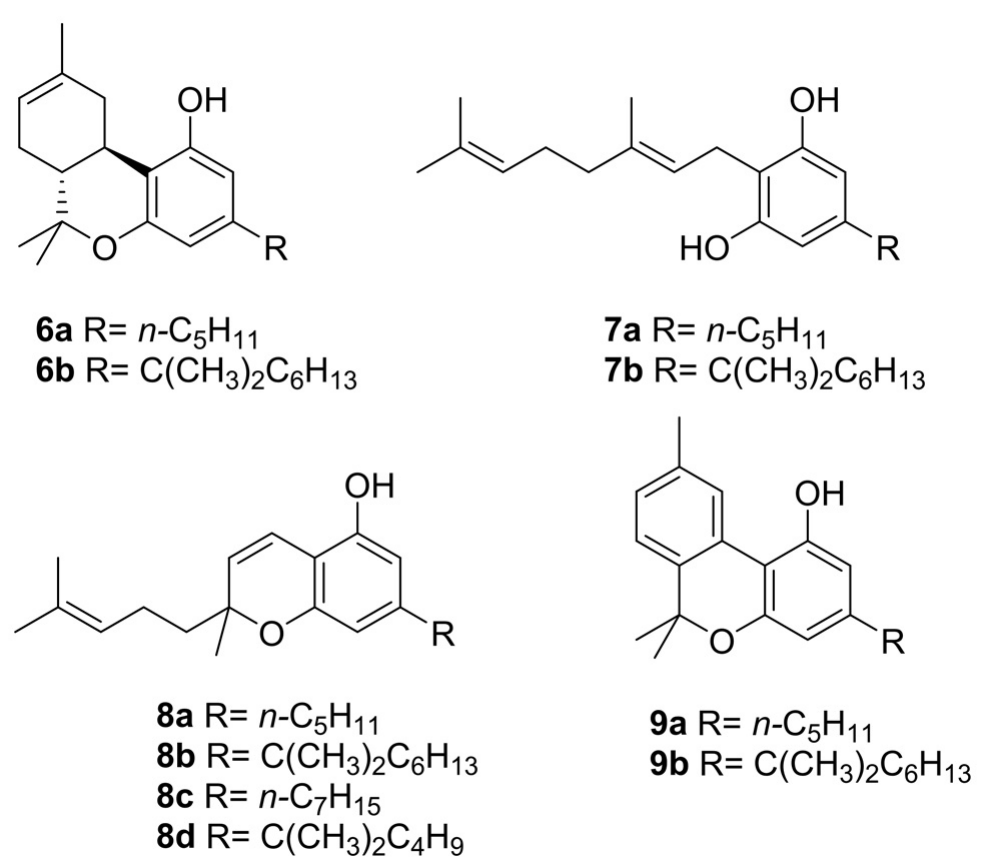
Structure of major phytocannabinoids Δ ^8^-THC (**6a**), CBG (**7a**), CBC (**8a**), and CBN (**9a**) and their α,α-dimethylheptyl analogues (**6b**–**9b**).

Phytocannabinoids are multi-target agents, capable of modulating a variety of end-points that includes not only metabotropic receptors (CB_1_, CB_2_), but also enzymes, nuclear receptors, and ionotropic receptors belonging to the family of thermo-TRPs (TRPV1-V4, TRPM8, TRPA1) [5]. In particular, thermo-TRPs represent relevant pharmacological targets in a wide array of pathological conditions ranging from pain to inflammatory and respiratory diseases [6], including those linked to COVID-19 [7]. Surprisingly, no systematic attempt has been made so far to investigate the effect of branching and elongation on the overall biological profile of phytocannabinoids. To fill this gap, we have synthesized the α,α-dimethylheptyl analogues of the five major phytocannabinoids (cannabidiol (CBD, **1a**), Δ^8^-tetrahydrocannabinol (Δ^8^-THC, Figure 2, **6a**) ( This biologically similar Δ^8^-analogue of **3** was used because of its major stability and ease of synthesis.), cannabigerol (CBG, **7a**), cannabichromene (CBC, **8a**), and cannabinol (CBN, **9a**)) and compared their profile of activity against thermo-TRPs with the one of their natural analogues.

## 2. Materials and Methods

### 2.1. Synthesis

#### 2.1.1. General Experimental Procedures

IR spectra were recorded on an Avatar 370 FT-IR Techno-Nicolet apparatus. ^1^H (400 MHz) and ^13^C (100 MHz) NMR spectra were measured on a Bruker Avance 400 MHz spectrometer. Chemical shifts were referenced to the residual solvent signal (CDCl_3_: δ_H_ = 7.21, δ_C_ = 77.0). Homonuclear ^1^H connectivities were determined by the correlation spectroscopy (COSY) experiment. One-bond heteronuclear ^1^H-^13^C connectivities were determined with the heteronuclear single quantum coherence (HSQC) spectroscopy experiment. Two-and three-bond ^1^H-^13^C connectivities were determined by gradient two-dimensional (2D) heteronuclear multiple bond correlation (HMBC) experiments optimized for a ^2, 3^ *J* = 9 Hz. Low- and high-resolution electrospray ionization mass spectrometry (ESI-MS) data were determined on an LTQ OrbitrapXL (Thermo Scientific) mass spectrometer. Reactions were monitored by thin-layer chromatography (TLC) on Merck 60 F254 (0.25 mm) plates, visualized by staining with 5% H_2_SO_4_ in EtOH and heating. Organic phases were dried with Na_2_SO_4_ before evaporation. Chemical reagents and solvents were purchased from Sigma-Aldrich, TCI Europe, or Fluorchem, and were used without further purification unless stated otherwise. Petroleum ether with a boiling point of 40–60 °C was used. Silica gel 60 (70–230 mesh) was used for gravity column chromatography (GCC). All work-up solutions were dried with Na_2_SO_4_ before evaporation.

#### 2.1.2. Depentyl-α,α-dimethylheptyl-cannabidiol (DMH-CBD, **1b**)

Under a nitrogen atmosphere, BF_3_OEt_2_ (1.5 mL/g substrate, 150 µL) was added to a stirred suspension of Al_2_O_3_ (10 g/g of substrate, 1 g) in dry DCM (10 mL). After stirring for 15 min at room temperature, the suspension was heated to 40 °C for 1 min, and then the resorcinol 10 (100 mg, 0.423 mmol) and (1*S*,4*R*)-*p*-mentha-2,8-dien-1-ol (11.52 mg, 0.34 mmol, 0.8 molar equiv.) were sequentially added. The reaction was stirred at 40 °C for 10 s. and then quenched with 5 mL of sat. Na_2_CO_3_. The resulting biphasic system was extracted with EtOAc, and the organic phase was washed with brine, dried, and concentrated under reduced pressure. Purification by flash column chromatography on silica gel (PE 100% to PE-EtOAc 95:5 as eluent) gave **1b** (94 mg, 60%, Rf: 0.75 (PE-EtOAc 9:1)) as a colorless oil. ^1^H NMR (400 MHz, CDCl_3_): δ 6.25–6.23 (br s, 2H), 5.90–6.05 (br s, 1H), 5.56 (s, 1H), 4.65 (s, 1H), 4.54 (s, 1H), 4.55 (s, 1H), 3.85 (br s, 1H), 2.30–2.05 (m, 2H), 1.79 (s, 3H), 1.63 (s, 3H), 1.45–1.50 (m, 2H), 1.21 (br s, 12 H), 0.95–1.05 (br s, 2H), 0.83 (t, *J* = 7.5 Hz, 3H); ^13^C NMR (100 MHz, CDCl_3_): δ 150.22, 149.5, 140.0, 124.1, 113.4, 110.7, 46.0, 44.6, 37.5, 37.3, 31.8, 30.4, 29.9, 28.7, 28.6, 28.4, 24.6, 23.6, 22.6, 20.7, 14.0; HRESIMS *m/z* [M + H]^+^ 371.2934 (calcd for C_25_H_39_O_2_, 371.2950).

#### 2.1.3. Depentyl-α,α-dimethylheptyl-Δ^8^-tetrahydrocannabinol (DMH-Δ^8^-THC, **6b**)

To a stirred suspension of 10 (450 mg, 1.69 mmol) in toluene (2.5 mL), p-toluensulfonic acid (PTSA, 60 mg, 0.34 mmol, 0.2 molar equiv.), and (1*S*,4*R*)-*p*-mentha-2,8-dien-1-ol (11,290 mg, 1.86 mmol, 1.1 molar equiv.) were sequentially added. The solution was heated at 120 °C for two hours and then cooled to room temperature and quenched with brine. The biphasic system was extracted with EtOAc, and the organic phase was dried and evaporated. Purification by flash column chromatography on silica gel (PE 100% to PE/DCM 8:2 as eluent) gave **6b** (532 mg, 85%, Rf: 0.90 (PE-EtOAc 9:1)) as a brown oil. ^1^H NMR (400 MHz, CDCl_3_): δ 6.42 (d, *J* = 1.8 Hz, 1H), 6.25 (d, *J* = 1.8 Hz, 1H), 5.49–5.41 (m, 1H), 4.79–4.71 (m, 1H), 3.22 (dd, *J* = 16.4, 4.4 Hz, 1H), 2.72 (td, *J* = 10.9, 4.7 Hz, 1H), 2.19–2.13 (m, 1H), 1.96–1.78 (m, 4H), 1.73 (s, 3H), 1.54–1.50 (m, 2H), 1.41 (s, 3H), 1.33–1.16 (m, 10H), 1.14 (s, 3H), 1.11–1.06 (m, 2H), 0.87 (t, *J* = 6.9 Hz, 3H); ^13^C NMR (100 MHz, CDCl_3_): δ 154.5, 154.45, 150.0, 134.7, 119.3, 110.1, 108.0, 105.4, 44.8, 44.4, 37.3, 36.0, 31.8, 31.5, 30.0, 28.7, 28.6, 27.9, 27.6, 24.6, 23.5, 22.6, 18.5, 14.1; HRESIMS *m/z* [M + H]^+^ 371.2934 (calcd for C_25_H_39_O_2_, 371.2950).

#### 2.1.4. Depentyl-α,α-dimethylheptyl-cannabigerol (DMH-CBG, **7b**)

Under a nitrogen atmosphere, BF_3_Et_2_O (1.5 mL/g substrate, 150 µL) was added to a stirred suspension of Al_2_O_3_ (10 g/g of substrate, 1 g) in dry DCM (10 mL). After stirring 15 min at room temperature, the suspension was heated to 40 °C for 1 min, and then the resorcinol 10 (100 mg, 0.42 mmol) and geraniol (12, 150 μL, 130 mg, 0.87 mmol, 2 mol. equivalents) were sequentially added. The reaction was stirred at 40 °C for 48 h, and then quenched with 20 mL of 2 *M* H_2_SO_4_. The reaction mixture was extracted with EtOAc, and the organic phase was washed with brine, dried, and concentrated under reduced pressure. Purification by flash column chromatography on silica gel (PE 100% to PE-EtOAc 95:5 as eluent) gave **1b** [94 mg, 60%, Rf: 0.75 (PE-EtOAc 9:1)] as a colorless oil. Purification by flash column chromatography on silica gel (PE 100% to PE-EtOAc 95:5 as eluent) gave **7b** (101 mg, 64%, Rf: 0.75 (PE-EtOAc 9:1)) as a colorless oil. ^1^H NMR (400 MHz, CDCl_3_): δ 6.40 (s, 2H), 5.32 (t, *J* = 6.6 Hz, 1H), 5.11 (br s, 2H), 5.08 (t, *J* = 6.7 Hz, 1H), 3.42 (d, *J* = 7.1 Hz, 2H), 2.21–2.07 (m, 4H), 1.84 (s, 3H), 1.70 (s, 3H), 1.62 (s, 3H), 1.55–1.51 (m, 2H), 1.29–1.16 (m, 12H) 1.12–1.05 (m, 2H), 0.87 (t, *J* = 6.9 Hz, 3H); ^13^C NMR (100 MHz, CDCl_3_): δ 154.5, 150.0, 139.0, 132.0, 123.7, 121.7, 110.2, 106.1, 44.4, 39.7, 37.4, 31.8, 30.0, 28.8, 26.4, 25.6, 24.6, 22.7, 22.3, 17.7, 16.2, 14.0; HRESIMS *m*/*z* [M + H]_+_ 373.3084 (calcd for C_25_H_41_O_2_, 373.3106).

#### 2.1.5. Depentyl-α,α-dimethylheptyl-cannabichromene (DMH-CBC, **8b**)

To a stirred suspension of 10 (350 mg, 1.48 mmol) in toluene (10 mL), *n*-butylamine (141 µL, 104 mg, 1.42 mmol, 1 molar equiv.) was added. The solution was heated to 60 °C for 10 min, then citral (13, 241 µL, 217 mg, 1.42 mmol, 1 molar equiv.) was added and the solution was refluxed overnight. The reaction was cooled to room temperature and quenched with 20 mL 2*M* H_2_SO_4_, and then extracted with EtOAc. The organic phase was washed with brine, dried, and concentrated under reduced pressure. Purification by flash column chromatography on silica gel (PE 100% to PE-EtOAc 95:5 as eluent) gave 210 mg **8b** (210 mg, 40%, Rf: 0.85 (PE-EtOAc 9:1)) as a brown oil. ^1^H NMR (400 MHz, CDCl_3_): δ 6.61 (d, *J* = 10.0 Hz, 1H), 6.39 (s, 1H), 6.25 (s, 1H), 5.51 (d, *J* = 10.0 Hz, 1H), 5.09 (t, *J* = 7.4 Hz, 1H), 2.32–2.02 (m, 2H), 1.76–1.62 (m, 2H), 1.65 (s, 3H), 1.57 (s, 3H), 1.55–1.44 (m, 2H), 1.39 (s, 3H), 1.31–1.12 (m, 6H), 1.19 (s, 6H), 1.11–0.99 (m, 2H), 0.94–0.60 (m, 3H); ^13^C NMR (100 MHz, CDCl_3_): δ 153.7, 152.0, 150.7, 131.6, 127.4, 124.1, 116.7, 107.0, 106.7, 105.5, 78.2, 44.4, 41.0, 37.7, 31.7, 30.0, 28.7, 26.2, 25.6, 24.6, 22.6, 17.6, 14.0; HRESIMS *m/z* [M + H]^+^ 371.2935 (calcd for C_25_H_39_O_2_, 371.2950). The same protocol was used for the preparation of **8c** and **8d**, starting, from 5-*n*-heptylresorcinol and 5-α,α-dimethylpentylresorcinol, respectively. Depentyl-5-*n*-heptylcannabichromene (**8c**): brown oil (42%, Rf: 0.87 (PE-EtOAc 9:1)). ^1^H NMR (400 MHz, CDCl_3_): δ 6.66 (d, *J* = 10.0 Hz, 1H), 6.26 (s, 1H), 6.16 (s, 1H), 5.50 (d, *J* = 10.0 Hz, 1H), 5.12 (t, *J* = 7.7 Hz, 1H), 2.45 (t, *J* = 7.9 Hz, 2H), 2.18–2.08 (m, 2H), 1.81–1.67 (m, 2H), 1.69 (s, 3H), 1.60 (s, 3H), 1. 59–1.50 (m, 2H), 1.40 (s, 3H), 1.37–1.16 (m, 8H), 0.90 (t, *J* = 6.9 Hz 3H); ^13^C NMR (100 MHz, CDCl_3_): δ 154.0, 151.3, 144.7, 131.6, 127.0, 124.2, 117.0, 108.9, 107.8, 107.1, 78.1, 41.0, 36.0, 31.8, 31.0, 29.3, 29.2, 26.2, 25.7, 22.7, 22.6, 17.6, 14.1; HRESIMS *m/z* [M + H]^+^ 346.2624 (calcd for C_23_H_35_O_2_, 343.2637). Depentyl-5-α,α-dimethylpentylcannabichromene (**8d**): brown oil [45%, Rf: 0.88 (PE-EtOAc 9:1)]. ^1^H NMR (400 MHz, CDCl_3_): δ 6.67 (d, *J* = 10.0 Hz, 1H), 6.42 (s, 1H), 6.31 (s, 1H), 5.52 (d, *J* = 10.0 Hz, 1H), 5.13 (t, *J* = 7.2 Hz, 1H), 2.18–2.12 (m, 2H), 1.80–1.65 (m, 2H), 1.69 (s, 3H), 1.55 (s, 3H), 1.56–1.49 (m, 2H), 1.43 (s, 3H), 1.27–1.19 (m, 2H), 1.24 (s, 6H), 1.13–1.03 (m, 2H), 0.85 (t, *J* = 7.3 Hz 3H); ^13^C NMR (100 MHz, CDCl_3_): δ 153.7, 151.9, 151.1, 131.6, 127.3, 124.2, 117.0, 106.8, 106.7, 105.7, 78.2, 46.8, 44.2, 41.0, 37.7, 35.4, 28.8, 26.9, 26.3, 25.7, 23.4, 22.7, 14.1; HRESIMS *m/z* [M + H]^+^ 343.2625 (calcd for C_23_H_35_O_2_, 343.2637).

#### 2.1.6. Depentyl-α,α-dimethylheptyl-cannabinol (DMH-CBN, **9b**)

To a stirred suspension of **8b** (210 mg, 0.57 mmol) in toluene (40 mL), iodine (291 mg, 1.15 mmol, 2 eq) was added. The solution was refluxed for three hours then cooled to room temperature and quenched with sat. Na_2_SO_3_. The mixture was extracted with EtOAc, dried, and concentrated under reduced pressure. Purification by flash column chromatography on silica gel (PE 100% to PE-CH_2_Cl_2_ 9:1 as eluent) gave **9b** (173 mg, 83%, Rf: 0.85 (PE-EtOAc 9:1)) as a brown oil. ^1^H NMR (400 MHz, CDCl_3_): δ 8.20 (s, 1H), 7.18 (d, *J* = 7.9 Hz, 1H), 7.10 (d, *J* = 8.9 Hz, 1H), 6.59 (d, *J* = 1.8 Hz, 1H), 6.44 (d, *J* = 1.8 Hz, 1H), 2.41 (s, 3H), 1.63 (s, 6H), 1.58–1.54 (m, 2H), 1.27 (s, 6H), 1.26–1.13 (m, 6H), 0.86 (t, *J* = 6.9 Hz, 3H); ^13^C NMR (100 MHz, CDCl_3_): δ 154.3, 152.7, 151.8, 136.9, 136.9, 127.6, 127.5, 126.3, 122.6, 108.6, 108.3, 107.6, 44.4, 37.6, 31.7, 29.9, 28.6, 27.1, 24.6, 22.6, 21.5, 14.0. HRESIMS *m/z* [M + H]+ 367.2623 (calcd for C_25_H_35_O_2_, 367.2637).

### 2.2. TRP Modulatory Activity

Effects of compounds on intracellular Ca^2+^ concentration ([Ca^2+^]_i_) were determined using Fluo-4, a selective intracellular fluorescent probe for Ca ^2+^. Assays of rat TRPA1, TRPV2, TRPV3, TRPV4, and TRPM8 or human TRPV1 mediated elevation of intracellular Ca ^2+^ in transfected HEK-293 cells were performed with a continuous monitoring of [Ca ^2+^]_i_ during the experiments [8]. Briefly, human embryonic kidney (HEK-293) cells, stably transfected with rat TRPA1, TRPV2, TRPV3, TRPV4, and TRPM8 or human TRPV1 (selected by geneticin 600 μg mL ^−1^) or not transfected, were cultured in EMEM+ 2 mM glutamine + 1% nonessential amino acids +10% FBS and maintained at 37 °C with 5% CO_2_. TRP-HEK-293 cells express stably high levels of TRP transcripts, while these transcripts were virtually absent in wild-type HEK-293 cells as checked by real-time PCR. On the day of the experiment, the cells were loaded for 1 h in the dark at room temperature with Fluo-4 AM (4 μM in DMSO containin 0.02% Pluronic F-127). The cells were rinsed, resuspended in Tyrode’s solution (145 mM NaCl, 2.5 mM KCl, 1.5 mM CaCl_2_, 1.2 mM MgCl_2_, 10 mM *D*-glucose, and 10 mM HEPES, pH 7.4), and transferred to a quartz cuvette of a spectrofluorimeter (PerkinElmer LS50B; λEX = 488 nm, λEM = 516 nm) equipped with a PTP-1 fluorescence Peltier system (PerkinElmer Life and Analytical Sciences, Waltham, MA, USA) under continuous stirring. Cell fluorescence before and after the addition of various concentrations of test compounds was measured, normalizing the effects against the response to ionomycin (IM, 4 μM). The values of the effect on [Ca^2+^]_i_ in HEK-293 cells not transfected were used as baseline and subtracted from the values obtained from transfected cells. For TRPA1, agonist efficacy was expressed as a percentage of the effect on [Ca ^2+^]_i_ observed with 100 μM allylisothiocyanate. The potency of the compounds (EC_50_ values) is determined as the concentration required to produce half-maximal increases in [Ca ^2+^]_i_. In the TRPV3 assay, TRPV3-expressing HEK293 cells were first sensitized with the structurally unrelated agonist 2-aminoethoxydiphenyl borate (100 μM). Antagonist/desensitizing behavior is evaluated against the agonist of the TRP analyzed by adding the compounds directly in the quartz cuvette 5 min before stimulation of cells with the agonist. Allylisothiocyanate (100 μM) was used for TRPA1, capsaicin (0.1 μM) for TRPV1, cannabidiol (2 μM) for TRPV2, thymol (100 μM) for TRPV3, GSK1016790A (10 nM) for TRPV4, and icilin (0.25 μM) for TRPM8. The IC_50_ value was expressed as the concentration exerting a half-maximal inhibition of agonist effect, taking as 100% the effect on [Ca^2+^]_i_ exerted by the agonist alone. Dose-response curve fitting (sigmoidal dose-response variable slope) and parameter estimation were performed with Graph-Pad Prism8 (GraphPad Software Inc., San Diego, CA, USA). All determinations were performed at least in triplicate.

### 2.3. Molecular Docking

Docking studies were performed with AutoDock 4.21 [9]. The rTRPA1 model, along with the ligands, were processed with AutoDock Tools (ADT) package version 1.5.6rc1 to merge non polar hydrogens, calculate Gasteiger charges, and select rotatable sidechain bonds. Grid dimensions of 60 × 60 × 50, centered in the putative binding pocket, were generated with the program AutoGrid 4.2 included in Auto-dock 4.2 distribution, with a spacing of 0.375 Å. Docking runs were carried out by either keeping the whole protein fixed or allowing the rotation of selected residues (Leu873, Leu884, Phe912, Met915 and Met949). A total of 100 molecular AutoDock docking runs for each docking calculation were performed adopting a Lamarckian genetic algorithm (LGA) and the following associated parameters: 100 individuals in a population with a maximum of 15 million energy evaluations and a maximum of 37,000 generations, followed by 300 iterations of Solis and Wets local search. Flexibility was used for all rotatable bonds of both docked ligands. The representative poses for each receptor were selected for the subsequent energy minimization with Amber16 package [10] using ff14SB force field for the protein, and gaff parameters for the ligand [11,12].

## 3. Results

### 3.1. Synthesis

**Scheme 1:**
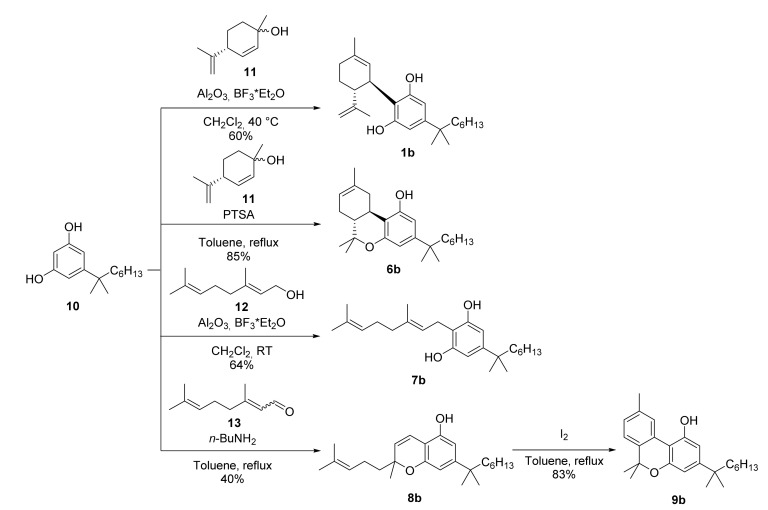
Synthesis of α,α-dimethylheptyl analogues of major phytocannabinoids DMH-CBD (**1b**), DMH-Δ^8^-THC (**6b**), DMH-CBG (**7b**), DMH-CBC (**8b**) and DMH-CBN (**9b**).

All α,α-DMH analogs were prepared from 5-(1,1-dimethylhepty)-resorcinol (**10**), in turn prepared from 3,5-dimethoxybenzoic acid [13]. Terpenylation was then carried out mutuating chemistry developed for the synthesis of the corresponding natural n-pentyl phytocannabinoids. C-Menthylation with (4R)-2,9-p-menthadien-1-ol (**11**) afforded either DMH-CBD (**1b**) or DMH-Δ^8^-THC (**6b**) depending on the reaction conditions [14], while geranylation with BF_3_ on alumina gave DMH-CBG (**7b**) [15], and chromenyation with citral (**13**) under basic conditions generated DMH-CBC (**8b**) [16], next aromatized to DMH-CBN (**9b**) by treatment with iodine (Scheme 1) [17].

### 3.2. Biological Evaluation

All DMH-derivatives retained the full agonistic profile on TRPA1 typical of their naturally occurring *n*-pentyl analogues, along with a comparable efficacy and ability to desensitize this channel (Table 1). Conversely, the replacement of the pentyl chain with the branched DMH group was detrimental for the activity toward TRPV1–4 and TRPM8 channels (Table 1 and Table 2). Thus, while CBD (**1a**) and CBG (**7a**) are TRPV1 antagonists in a sub/low micromolar range, their correspondent DMH derivatives (**1b** and **7b**, respectively) were inactive against this receptor. Similarly, while the natural derivatives are antagonists in a low micromolar range at TRPV4, the activity was lost for all DMH-derivatives, that were only weak inhibitors of TRPV2, with IC_50_ ranging from 9 to >50 μM. As for TRPV3, DMH-Δ^8^-THC (**6b**) was the only compound active as an inhibitor at a low micromolar range (IC_50_ of ~2 μM), with a slightly better potency than Δ^8^-THC (**6a**). At TRPM8, al DMH analogues acted as pure antagonists albeit with a lower potency than the natural derivatives, except DMH-CBC (**8b**), which was totally inactive, and with only DMH-CBN (**9b**) retaining a certain affinity (IC_50_~1 μM).

To better rationalize the selectivity of the DMH-derivatives toward TRPA1 and dissect the relative contribution of chain elongation and branching, two point-mutated analogues of the most potent compound (DMH-CBC (**8b**)) were prepared according to the general synthetic sequence summarized in Scheme 1 starting from a different 5-alkylresorcinol. In the *n*-heptyl chromene **8c**, the alkyl chain is linear and expanded by two carbon atoms compared to CBC (**8a**), while in the α,α-dimethylpentyl analogue **8d** the benzyl branching is implanted on a pentyl chain. As shown in Table 1, the *n*-heptyl derivative **8c** was less potent than the dimethyl-pentyl analogue **8d** (IC_50_ = 3.8 and 0.40 μM, respectively). Hence, the α,α-dimethyl substitution is the major contributor to the activity of the DHM derivative (IC_50_ = 0.32 μM).

### 3.3. Molecular Docking Studies on CBC and CBC-DMH at rTRPA1

**Figure 3:**
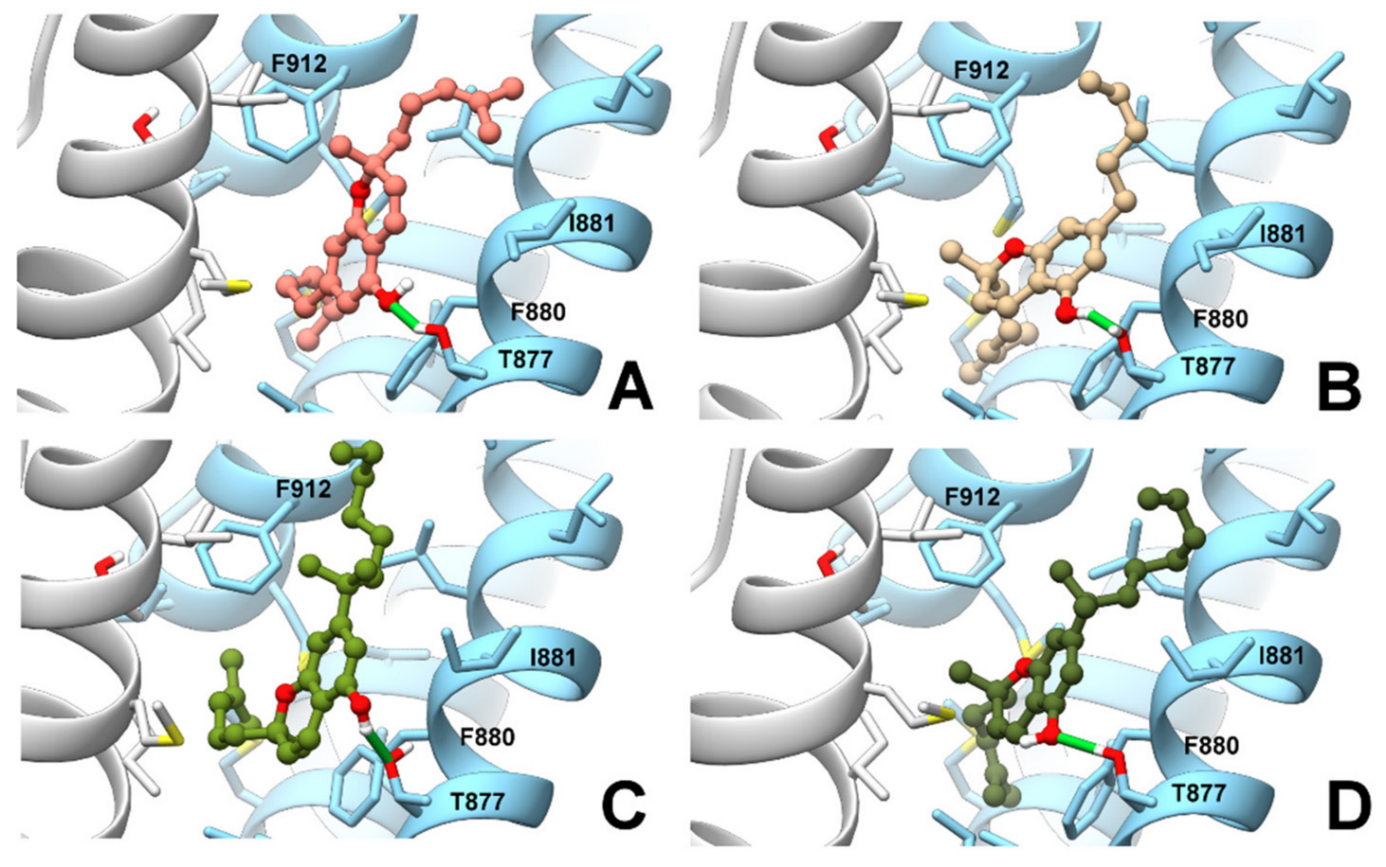
Representative complexes of the rTRPA1 model with (R)-CBC (colored in salmon, panel (**A**)), (S)-CBC (colored in tan, panel (**B**)), (R)-CBC-DMH (colored olive drab, panel (**C**)) and (S)-CBC-DMH (colored in dark green, panel (**D**)). A ribbon representation is used for the protein backbone and sticks for protein side chains of residues within 5 Å from the ligand, in ball & stick representation. H-bonds are shown as green sticks. Carbon atoms are painted according to receptor subunits. Sulfur, oxygen, and polar hydrogen atoms are painted yellow, red, and white, respectively.

To shed light on the putative binding modes of DMH-CBC (**8a**) on TRPA1 channels, a molecular docking study was carried out using our previous homology model of ratTRPA1 [20]. Since the compound is a racemate, both enantiomers were considered, using CBC (**8a**) as a reference compound. Different docking runs were carried out by either keeping the protein side chains rigid or allowing flexibility for selected residues, as described in detail in the Materials and Methods section. The best docking poses for both CBC and DMH-CBC, selected on the basis of binding energy value and visual inspection, were subjected to energy minimization (Figure 3). Both enantiomers of CBC and DMH-CBC engage H-bonding with their phenolic hydroxyl and Thr877 (monB). (*R*)-CBC points its pentyl chain in a hydrophobic cleft formed by Ile953 (monA), Met956 (monB), and Phe880 (monB), while the methyl group of the pentyl chain forms a CH-π interaction with Phe880 and the chromene methyl is surrounded by the hydrophobic residues Ile881 (monB), Leu885 (monB), Phe912 (monB), and Ile945 (monA). Conversely, (*S*)-CBC adopts a flipped orientation with the pentyl chain now pointing toward Phe912 (monB), engaging a CH-π interaction with the aromatic ring of this amino acid. The same arrangement of (*S*)-CBC is adopted by both enantiomers of CBC-DMH (**8b**), with the gem-dimethyls sandwiched between Phe912 (monB) and Ile881 (mon B). In this arrangement, the alkyl chain stabilizes the complex by hydrophobic interactions, counteracting the higher flexibility of the heptyl chain in comparison with the pentyl one and in accordance with the lower activity of the *n*-heptyl chain analogue, with the length of the CBC pentyl chain being consistent with alternative CH-π interaction with Phe880/Phe912 within the binding pocket. The two DMH-CBC enantiomers share the same orientation of the DMH-chain but show a distinct orientation of the chromene methyl group: in the *R* enantiomer, the methyl group points toward Phe880, and the terpenoid chain is hosted in a hydrophobic cleft at the interfaces between two monomers. Conversely, in the *S* enantiomer the orientation of these group is just the opposite.

## 4. Discussion

The serendipitous discovery by Adams that a combination of branching and elongation of the alkyl chain boosts the narcotic potency of tetrahydrocannabinols provided the basis for the synthesis of highly potent molecular probes, eventually leading to the characterization of the two cannabinoid receptors (CB1 and CB2) and to the synthesis of nabilone (Cesamet), a clinically efficacious synthetic analogue of Δ^9^-THC [2]. Despite the relevance of this maneuver for CB_1_ and CB_2_ affinity, and its potential to bias activity toward specific targets, its effect has not yet been investigated on other high-affinity targets of phytocannabinoids like the thermo-TRPs (TRPV1-V4, TRPM8 and TRPA1) [5]. This is surprising, since one major issue plaguing the bench-to-bed translation of the biological activity of cannabinoids is their promiscuous end-point profile, which spans metabotropic and ionotropic receptors as well as enzymes and transcription factors. For Δ^9^-THC, the interaction with the two cannabinoid receptors dominates, albeit not fully recapping, the clinical profile, while the other phytocannabinoids show a constellation of end-points characterized by a generally modest affinity for macromolecular end-points, with sub-micromolar potency basically limited to some thermo-TRPs [5].

To systematically address this issue, we have synthesized the α,α-dimethylheptyl analogues of the five major phytocannabinoids and have comparatively evaluated their activity on thermo-TRPs. The results showed that, by quenching affinity for the other thermo-TRPs, the replacement of the linear pentyl substituent with an α,α-dimethylheptyl residue induces a selectivity switch toward TRPA1 in all major phytocannabinoid chemotypes. Molecular docking studies rationalized these results in terms of binding, capitalizing our previously developed homology model of ratTRPA1 [20]. Structural and mutagenic experiments have identified two binding sites for non-electrophilic agonists, centered, respectively, on hThr874 (rThr877, uniprot ID:Q6RI86, Site 1) and hTyr840 (Site 2). However, only Site1 was considered, since, unlike phytocannabinoids and their DMH analogues, the selective Site 2 agonist GNE551 lacks channel desensitizing properties. Moreover, Tyr840, critical for GNE551 binding, is not conserved between human and rat hortologues, being replaced by a Phe residue in the latter. The results of the docking studies suggest the *gem*-dimethyls on the benzyl carbon provide an additional anchoring site to engage the binding pocket into non-covalent interactions, rationalizing the retention of affinity for TRPA1. This maneuver is detrimental for the other thermos-TRPs presumably because of the incapacity to accommodate the steric and lipophilicity changes associated with benzyl branching into less broadly tuned binding sites [21].

Non-covalent modulators of TRPA1 are relatively few compared to the covalent ones [22], and their biological profile is critically dependent on their binding mode. Site 2 ligands like GNE551 do not elicit channel desensitization and tachyphylaxis, thus inducing persistent pain insensitive to TRPA1 antagonists [23]. Conversely, Site 1 ligands induce only transient pain, followed by desensitization and analgesia [24]. The desensitization of TRPA1 has the potential to translate into beneficial effects for a wide range of inflammatory conditions [25], traditionally focused in the realm of inflammation and pain [20]. However, growing evidence of involvement in the SARS-CoV-2 infection through the modulation of inflammation, pain, and fever, has provided an additional therapeutic area of investigation, with documented proof-of-concept clinical cases of the beneficial effects of sulforafane, a TRPA1 and Nrf2-interacting nutrient, on the course of the infection [7].

## 5. Conclusions

Replacement of the *n*-pentyl side chain with an α,α-dimethylheptyl group quenches the exuberant interaction of phytocannabinoids with thermo-TRPs, making them selective non-covalent modulators of TRPA1. Within the DMH-analogues of the most common phytocannabinoids, DMH-CBC (**8b**) emerged as the most potent compound in the series, and the molecular details of its interaction with this ion channel were investigated and compared to those of the parent compound CBC (**8a**). Both compounds are racemic, but only the enantiomers of the natural product CBC showed a distinct orientation at TRPA1 binding sites. These results provide a basis for a broader comparison that will also include additional end points of phytocannabinoids and their endogenous analogues, like anandamide and palmitoylethanolamide.

## Figures and Tables

**Table 1:** Efficacy, potency, and inhibitory effect on TRPA1, TRPV1, and TRPM8 channels.

	TRPA1			TRPV1			TRPM8
	Efficacy (%ITC 100 μM)	Potency EC_50_ μM	IC_50_^a^ TRPA1 μM	Efficacy (% IM 4 μM)	Potency EC_50_	IC_50_^b^ TRPV1 μM	IC_50_^c^ TRPM8 μM
**1b** **1a** [18]	133.4 ± 5.3 115.9 ± 4.6	0.40 ± 0.15 0.11 ± 0.05	0.51 ± 0.03 0.16 ± 0.05	<10 44.7 ± 0.02	NA 1.0 ± 0.1	>100 0.6 ± 0.05	13.4 ± 2.1 0.06 ± 0.01
**6b** **6a** [18]	124.2 ± 4.0 117.0 ± 12.0	4.8 ± 1.1 0.23 ± 0.03	6.0 ± 0.9	15.3 <10	>100 NA	>100	39.9 ± 6.9 0.16 ± 0.01
**9b** **9a** [18]	132.3 ± 7.9 83.3 ± 12.0	2.1 ± 0.9 0.18 ± 0.02	3.2 ± 0.6 0.40 ± 0.04	<10 <10	NA	>100 >50	0.98 ± 0.12 0.21 ± 0.05
**7b** **7a** [18]	99.2 ± 4.5 99.9 ± 1.1	9.1 ± 2.0 0.70 ± 0.03	1.7 ± 0.15 13.0 ± 4.8	<10 33.8 ± 2.3	NA 1.3 ± 0.5	>100 2.6 ± 0.5	9.2 ± 0.8 0.16 ± 0.02
**8b** **8a** [18]	120.4 ± 2.8 119.4 ± 3.1	0.76 ± 0.12 0.09 ± 0.01	0.32 ± 0.01 0.37 ± 0.05	18.9 ± 0.2 <10	12.8 ± 0.9	>100 >50	>100 40.7 ± 0.6
**8c**	97.8 ± 3.3	1.2 ± 0.2	3.8 ± 0.35	<10	NA	>100	>100
**8d**	98.7 ± 1.9	0.15 ± 0.03	0.40 ± 0.01	<10	NA	>100	23.6 ± 7.4

Reference agonists: ^a^ AITC 100 μM; ^b^ Capsaicin 0.1 μM; ^c^ Icilin 0.25 μM.

**Table 2:** Efficacy, potency, and inhibitory effect on TRPA1, TRPV1, and TRPM8 channels.

	TRPV2			TRPV3			TRPV4		
	Efficacy (% IM 4 μM)	Potency EC_50_ μM	IC_50_^a^ TRPV2 μM	Efficacy (% IM 4 μM)	Potency EC_50_ μM	IC_50_^c^ TRPV3 μM	Efficacy (% IM 4 μM)	Potency EC_50_ μM	IC_50_^e^ TRPV4 μM
**1b** **1a** [18,19]	<10 40.5 ± 1.6	NA 1.25 ± 0.23	16.8 ± 0.2 4.5 ± 0.7 ^b^	15.8 ± 0.4 50.1 ± 4.8	11.0 ± 1.1 3.7 ± 1.6	32.6 ± 5.1 0.9 ± 0.3 ^d^	34.0 ± 1.3 16.7 ± 1.0	NA 0.8 ± 0.3	>100 1.1 ± 0.1 ^f^
**6b** **6a** [18,19]	<10 53.0 ± 1.4	NA 0.65 ± 0.05	45.8 ± 3.9 0.8 ± 0.01	53.8 ± 1.6 18.2 ± 1.0	0.14 ± 0.03 9.5 ± 1.9	2.1 ± 0.6 32.3 ± 2.1	<10 <10	NA NA	>100 15.2 ± 2.7
**6b** **6a** [18,19]	10.5 ± 0.02 39.9 ± 2.1	0.25 ± 0.03 19.0 ± 3.7	>50 15.7 ± 2.1	20.9 ± 3.0 13.1 ± 2.4	41.4 ± 21.6 5.3 ± 2.7	>100 9.4 ± 0.1	12.1 ± 0.2 15.3 ± 1.5	16.1 ± 21.6 16.1 ± 4.5	>100 5.4 ± 0.8
**7b** **7a** [18,19]	65.3 ± 0.7 73.6 ± 1.2	9.8 ±0.6 1.7 ± 0.08	39.4 ± 4.5 1.5 ± 0.2	64.6 ± 4.8 18.5 ± 0.7	39.7 ± 0.03 1.0 ± 0.2	35.4 ± 3.6 >50	34.4 ± 5.2 23.7 ± 1.8	39.0 ± 21.6 5.1 ± 1.6	35.4 ± 3.2 1.3 ± 0.1
**8b** **8a** [18,19]	76.1 ± 0.6 <10	>50 NA	22.6 ± 0.2 6.5 ± 1.6	41.6 ± 1.8 20.2 ± 0.4	>50 1.9 ± 0.2	>50 >100	<10 22.9 ± 1.2	NA 0.6 ± 0.2	>100 9.9 ± 1.2
**8c**	<10	NA	9.0± 0.8	<10	NA	>100	<10	NA	>100
**8d**	<10	NA	13.3± 1.1	<10	NA	>100	<10	NA	>100

Reference agonists: ^a^ CBD 2 μM; ^b^ LPC 3 μM; ^c^ Thymol 100 μM; ^d^ Carvacrol 1 mM; ^e^ GSK1016790A 10 nM; ^f^ 4αPDD 1 μM.

## Data Availability

Not applicable.
